# Transplant tourism among kidney transplant patients in Eastern Nigeria

**DOI:** 10.1186/s12882-017-0635-1

**Published:** 2017-07-05

**Authors:** U. H. Okafor

**Affiliations:** Renal Unit, Department of Medicine, Enugu State University Teaching Hospital, Parklane Enugu, Enugu, State Nigeria

## Abstract

**Background:**

Transplant tourism entails movement of recipient, donor or both to a transplant centre outside their country of residence. This has been reported in many countries; and has variously been associated with organ trade.

The objective of this study is to determine the frequency and pattern of transplant tourism among transplant patients in Eastern Nigeria.

**Methods:**

This is a non randomized cross sectional study. All kidney transplant patients who presented at Enugu State University Teaching Hospital Parklane Enugu and Hilton Clinics Port Harcourt in Nigeria were recruited. The clinical parameters including the transplant details of all the patients were documented. The data obtained was analysed using SPSS package.

**Results:**

A total of one hundred and twenty six patients were studied, 76.2% were males with M:F ratio of 3.2:1 and mean age of 46.9 ± 13.3 years. Fifty four and 58.7% of the patients were managed in a tertiary hospital and by a nephrologist respectively before referral for kidney transplant. Only 15.8% of the patients had their kidney transplant without delay: finance, lack of donor, logistics including delay in obtaining travelling documents were the common causes of the delay.

Ninety percent of the patients had their transplant in India with majority of them using commercial donors. India was also the country with cheapest cost ($18,000.00). 69.8% were unrelated donors, 68.2% were commercial donors and 1.6% of the donors were spouse. All the commercial donors received financial incentives and each commercial donor received mean of 7580 ± 1280 dollars. Also 30.2% of the related donors demanded financial incentive.

**Conclusion:**

Transplant tourism is prevalent in eastern Nigeria.

## Background

A significant new element of a growing trade in healthcare has involved the movement of patients across borders in the pursuit of medical treatment and health; a phenomenon commonly termed medical tourism. This medical treatment span the full ranges of medical services, however surgical procedures including organ transplant is among the major treatment undertaken by patients abroad.

This trend in movement of patients and occasionally medical personnel across border has brought succor to patients who hitherto couldn’t have accessed the medical treatment.

Thus transplant (organ) tourism entails movement of organ, donor or recipients across border [1]. Yosuke Shimazono illustrates four modes of transplant tourism [2]

1. Recipient travelling to country where transplant centre and donor is located.
2. Donor travelling to country where the recipient and transplant centre is located.
3. Donor and recipient travelling together to country where the transplant centre is located.
4. Donor and recipient travelling from different countries to another country where the transplant centre is located.

Transplant tourism though can be legal, appropriate and permissible; it has been a leeway to various unethical activities including organ trafficking and bad medical practices. Thus World Health Organization (WHO) argues that though transplantation promotes health but the notion of “transplantation tourism” has the potential to violate human rights or exploit the poor. This could lead to unintended health consequences and unequal access to services; which ultimately may cause harm. Consequently a summit convened by The Transplantation Society (TTS) and the International Society of Nephrology (ISN) [3] in 2008 at Istanbul in partnership with many countries through various acts, decrees and edict has discouraged commercial transplant tourism/organ trafficking (Istanbul declaration).

Transplant tourism has been reported in many countries with destinations to India, Pakistan, Philippines, Egypt, China, Mexico etc., [1, 4, 5] however there are no precise data on the frequency of transplant tourism/organ trafficking in these countries. Organ trafficking has been estimated to accounts for 5–10% of annual kidney transplants globally [2].

In Nigeria [6] there is a growing population of patients with kidney transplant and transplant centres, however only 2 of the 8 centres that had done kidney transplant are transplanting regularly. A report from the most active transplant centre in Nigeria by Ebun Bamgboye [7] at the biennial satellite symposium of the World congress of nephrology (10th conference on kidney disease in disadvantaged population) in Cape Town South Africa 2015 showed that more than 50% of their kidney transplant population had kidney their transplant abroad.

In the national health act [8] which was recently signed into law in Nigeria, it is an offence for a donor or any of its agent to sell, trade or receive any financial or other reward for such donation, except for the reimbursement of reasonable costs incurred by him or her to provide such donation. Thus commercial donation and transplant trafficking is an offence in Nigeria punishable by imprisonment and/or option of fine. Hitherto there is no study on transplant tourism/commercial kidney donation in Nigeria.

This study is aimed to determine the frequency of transplant tourism and commercial organ donation among kidney transplant patients in south eastern Nigeria.

## Methods

This is a cross sectional non randomized study.

### Study location

The study locations were Hilton Clinics Port Harcourt Rivers state and ESUT Teaching Hospital (ESUTH) Enugu in Enugu state, both located in the Eastern region of Nigeria. Each centre has 5 haemodialysis machines; there is no facility for peritoneal dialysis in both centres. A total of about 250 haemodialysis sessions is done in both centres (170 in Hilton clinics and 80 in ESUTH) monthly. A total of about 50 patients are dialysed monthly with majority unable to sustain regular maintenance haemodialysis.

Nigeria was divided by colonial rulers in 1914 into 3 geographical regions of north, west and east. Each of the regions has diverse cultural, social, religious, and economic characteristics. The country was on various later occasions divided into various states for better administration; and currently made up of 36 states and the federal capital territory.

The then eastern region is located in the eastern part of the country and currently has 9 states. These states are Abia, Akwa Ibom, Anambra, Bayelsa, Cross rivers, Ebonyi, Enugu, Imo, and Rivers states. They are heterogeneous multilingual enclave, predominantly Christians, domain to the oil communities in Nigeria. It is also centre of agrarian and commercial activities. The 2 study locations are situated in the northern (Enugu) and southern (Rivers) state in the region. The hospitals attend to patients with kidney diseases from the states in the region and beyond.

### Study population

These are adult patients with end stage kidney disease presenting at the study locations between 1^st^ January 2008 and 31^st^ December 2015; who have either had kidney transplant, or whose kidney transplant was aborted after full pre transplant work up including securing a donor, transplant location and the tariff was completed. Ethical clearance was obtained from the ethical and research committee of the Hospitals.

The details of the study were explained to the patients/caregivers and informed consent obtained from each of the patient before recruiting them for the study. Consecutive patients who gave consent and fulfilled the inclusion criteria were recruited.

### Data collection

The biodata, clinical characteristics, location of the transplant centre, details of the donor and cost of the kidney transplant were obtained from the patients, care givers and hospital records of the transplant centre.

### Data analysis

The data obtained were entered into an excel spread sheet, and analysed using statistical package for social sciences version 17 (IBM New York USA). The data were presented as frequencies, means and standard deviations.

## Results

### Epidemiologic data

A total of 169 patients seen during the study period in these hospitals met the inclusion criteria; however 126 patients (74.6%) had complete information. Ninety three patients (73.8%) had transplant and 33 patients (26.2%) had their transplant aborted during pre transplant work up in the transplant centres. Reasons for aborting transplant included demise of patients, complications of investigations, contraindications to transplant and donor withdrawal etc.

There were 96 males (76.2%) and 30 females (23.8%), with M:F of 3.2:1. The age range was 25 to 75 years and mean of 46.9 ± 13.3 years. Fifty two (41.3%) patients were public servants with 71% of them in the senior cadre, 30 patients (23.8%) were business men, 18 patients (14.3%) were house wives, 12 patients (9.5%) were students, 10 patients (8.0%) were clergy and 3 patients (2.4%) retiree. The Ijaws (52.4%) and the Ibos (42.9%) constitute the major ethnic group in the study (Table 1).

### Kidney disease

Diagnosis of the kidney disease was made in a tertiary health facility in 54% patients, in secondary health facility in 44.4% patients and primary health facility in 1.6% patients. Seventy four (58.7%) patients were managed and worked up for kidney transplant by a nephrologist, 17.5% patients by internist and 23.8% patients by general practitioners (medical officers). The common kidney diseases were chronic glomerulonephritis (32.5%), hypertensive nephrosclerosis (23%), diabetic nephropathy (11.1%) and HIV associated nephropathy (6.3%). The cause of kidney diseases was not known in 17.5% of the patients. Majority of the patients (98.4%) were on maintenance haemodialysis before the transplant (Table 1).

**Table 1:** Epidemiologic parameters

Parameters	No (126)	Percent (100)
Age distribution		
20–29	15	11.9
30–39	27	21.4
40–49	26	20.6
50–59	27	21.4
60–69	28	22.2
≥ 70	2	1.6
Sex		
Male	96	76.2
Female	30	23.8
Occupation		
Public servant	52	41.3
House wives	18	14.3
Business	30	23.8
Clergy	10	7.9
Students	5	4.0
Unemployed	7	5.6
Retiree	4	3.2
Renal diseases		
Chronic glomerulonephritits	41	32.5
Hypertensive nephrosclerosis	29	23
Lupus nephritis	2	1.6
Hivan	8	6.3
Diabetic nephropathy	14	11.1
ADPKD		
Sickle cell nephropathy	2	1.6
Toxic nephropathy	1	0.8
Analgesic nephropathy	2	1.6
Pre ecclampsia	2	1.6
Kidney allograft failure	1	1.6
Unknown	2	1.6
	22	17.5
Ethnicity		
Ijaw	66	52.4
Ibo	54	19
Yoruba	4	3.2
Hausa	2	1.6
Pretransplant dialysis		
Haemodialysis	124	98.4
Peritoneal dialysis	0	0
No dialysis	2	1.6
Pretransplant care		
Primary care practictioners	30	23.8
Internist	22	17.5
Nephrologist	74	58.7

### Kidney transplant

Only 15.8% of the patients were transplanted without difficulty; however causes of delay included finance in 36.5%, lack of donor in 19.0%, logistics in 22.2% and medical complications/comorbidities in 6.3%.

**Fig. 1:**
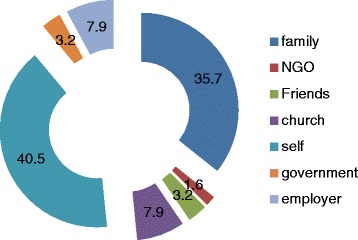
Sponsors of the kidney transplant

The kidney transplant was funded mainly by the patients and their families. The detail of the sponsors of the transplant is as documented in Fig. 1.

**Fig. 2:**
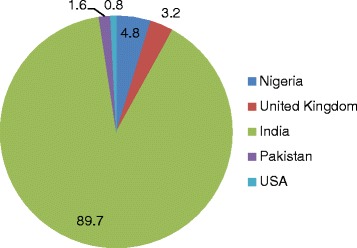
Countries of kidney transplant

About 90% of the patients had/planned to have their transplant in India while only 3.2% in Nigeria. The distribution of the countries where the patients had/intended to have the transplant is as documented in Fig. 2.

The donors were all living with 88 (69.8%) of them unrelated, 2 (1.6%) of them were spousal donors and 86 (68.2%) of them commercial donors.

All the commercial donors received financial reward; mean amount received by each commercial donor was 7580 ± 1280 dollars. Other rewards donors received include payment of rent in 1.6%, sponsoring of marriage in 0.8% and vacation in 1.6%.

Furthermore 13(10.3%) of the related donors (30.2%) were not altruistic as they demanded financial incentive. The distribution of the transplant centre and the type of donors is documented in Table 2.

**Table 2:** Distribution of type of donors and the transplant centres

Transplant centre (total no of transplant)	Related donor (%)	Unrelated donor (%)	Reward (%)	Agents (%)
Nigeria [6]	3 (50%)	3 (50%)	3 (50%)	3 (50%)
United kingdom [4]	4 (100%)	0 (0%)	0 (0%)	0 (0%)
India (113)	30 (26.5%)	83 (73.5%)	92 (81.4)	82 (72.6%)
Pakistan [2]	0 (0%)	2 (100%)	2 (100%)	2 (100%)
USA [1]	1 (100%)	0 (%)	0 (0%)	0 (0%)
Total (126)	38 (30.6%)	88 (69.4%)	97 (77%)	87 (69%)

The approximate cost of kidney transplant was 18,000 dollars in India, 32,000 dollars in Nigeria (most active centre), 78,000 dollars in UK and 117,000 dollars in USA.

## Discussion

This study revealed that the majority of the patients were males, middle aged and of low/middle socioeconomic class. This compares with a previous study in eastern Nigeria that reported that end stage kidney disease is commoner in males, middle age, and low/middle income class [9]. Furthermore a study of patients with kidney transplant in Nigeria revealed similar demographic pattern [10]. This preponderance of these characteristics has been attributed to various genetic and environmental factors.

Kidney diseases resulting or leading to collapse of kidney function is associated with marked morbidity and ultimate fatality if not managed appropriately. Thus it needs the highest form of available expertise and facility to improve the chances of obtaining the best possible outcome. The health facilities in most developing countries including Nigeria are very poor and fall short of recommended best practices guidelines. This study revealed that about 50% of the patients neither accessed a tertiary health facility nor were they managed by a nephrologist before referral for kidney transplant. Management of kidney diseases in resource poor nations are burdened by various economic, social, political and geographical challenges [11]. There are few tertiary health centers, nephrologists and other support facilities in Nigeria, with most of them located in the urban areas. However even where the facilities are available they are poorly utilized by the patients because of ignorance and/or poverty. These obstacles could have contributed in many of the patients in this study being managed by medical practitioners poorly equipped with knowledge of renal medicine. Thus these patients may not have received the best available pre transplant care.

Management of chronic kidney disease includes conservative treatment aiming at the correction of anaemia and the control of blood pressure, electrolyte, blood glucose, fluid, nutrition and infection. Renal replacement therapy (dialysis and kidney transplant) becomes inevitable as the kidney function deteriorates and disease progresses to the end stage. Kidney transplant is not yet prevalent in Nigeria [6]; only about 15% of the study population had the transplant without difficulty. The various factors that contributed to a delay in the transplant in this study include finance as patients had to sell or mortgage property and/or seek for a sponsor. Most sponsors were mainly relatives, religious organizations and rarely government/non - governmental organizations. This is in contrast to what is obtainable in most developed countries where the health insurance and other health management organizations sponsor kidney transplant. Other factors that delayed kidney transplant in these patients were difficulty in getting atruistic donors, complications of end stage kidney disease, co-morbidities and logistics including issuance of visa by the transplant country.

All the patients in this study are Nigerians residing in Nigeria, however only about 5% of the patients either had or planned to have their kidney transplant in centres within Nigeria. Thus the prevalence of transplant tourism in this study is about 95%. India was the major country of destination accounting for about 90% of the patients, and the United States of America (0.8%) was the least chosen country; United Kingdom and Pakistan were the other transplant tourism sites in this study. India has been noted as a country of destination for transplant/medical tourism for patients in Nigeria with media reports of the rampant trips to India by various social strata in the country for diverse medical treatments. India is also the transplant destination of patients from many countries of Asia and Europe [1, 4, 12]. The increase in transplant tourism to India started during the era when commercial transplantation was still tolerated. However though commercial organ donation has been outlawed since 1994 [13], India still remains the main port of kidney transplant for many overseas patients including the patients in this study. However the study by Adamu et [14] al reported that Pakistan was the major country of destination of transplant tourism, with China, Philippines and Egypt being the other countries of destinations of kidney transplant patients. Other major tourism destinations include Malaysia, Singapore, Thailand, South Africa, Brazil, Costa Rica, Cuba, Mexico, the Middle East, and a range of European destinations [1, 2, 5, 14]. The factors determining a patient’s decision to travel to a specific destination for kidney transplant include expertise, cost, donor approval, geographical proximity, advertisement and ease of obtaining travel documents from the country of destination [15]. As noted in this study more than 70% of kidney transplant done in India were from commercial donors and India was also noted to have the lowest tariff for kidney transplant. These could explain the choice of the majority of patients preferring India rather than the local transplant centers in Nigeria. Heavy reliance on overseas transplantation has been reported in many countries of Asia and Middle East [12].

Cadaveric kidney transplant has increased the prevalence of kidney transplantation remarkably [16–18]. However the shortage of organ leading to long waiting time is still prevalent with more than 50% of patients unable to receive a kidney transplant. None of the patients in this study had a transplant from a cadaveric donor or intended to have a transplant from a cadaveric donor. About 70% of the donors were unrelated to the patients and two third of them were commercial donors. This is in clear contradiction with the declaration in Istanbul [3] in 2008 that recommends altruistic donation and discourages any form of inducement that will compel a donor to sell an organ. The WHO has estimated that up to 10% of all organ transplants globally were of commercial origin by 2005 [1, 12, 19].

The recently promulgated National health act in Nigeria in 2014 [8] states that “It is an offence for a person who has donated tissue, blood or a blood product to receive any form of financial or other reward for such donation, except for the reimbursement of reasonable costs incurred by him or her to provide such donation; and to sell or trade in tissue, blood or blood products, except as provided for in this Act. Any person found guilty of this offence is liable on conviction to a fine of one hundred thousand naira(500 dollars) or to imprisonment for a period not exceeding one year or to both fine and imprisonment”. The effectiveness of this law to abolish or retard this noisome organ trade in Nigeria is in doubt. The impoverished people will readily part with their kidneys for money. The laxity in implementing the law and the ignorance on the part of the managing physicians (mostly non nephrologist) may still allow these activities of organ trafficking. If the act is not properly implemented, the middle men may device means of thwarting the good intent of this act.

The tendency to resort to transplant tourism among these end stage kidney transplant patients as reported in this study could have accrued from paucity of transplant centers in Nigeria and overwhelming ignorance in the population. Thus the need for government, industries and other non-governmental organizations to invest in the development of adequate health facilities to cater for the health need of the populace including management of patients with renal diseases. Policy makers should regulate treatment abroad and limit it to only for those treatments not available in the country. There should be massive awareness campaign to encourage altruistic kidney donation and discourage commercial organ donation. Public focused governance to improve the well being and economic state of the populace, as this will reduce the frequency of organ sale. In Iran, a realistic and community acceptable policy was developed in order to regulate living kidney donation; however appropriate application of this policy in developing countries is questionable [20].

## Conclusion

In conclusion the science of organ transplant is a tremendous development and achievement in human history. This must be allowed to flourish within strict ethical guidelines. This study revealed that transplant tourism and commercial organ donation is very prevalent among patients in Nigeria requiring kidney transplant.

The study had some limitations. It is a cross sectional study and thus unable to report the outcome of the patients. The small sample size is small. Notwithstanding the study has revealed a need for public enlightenment and formulation of policies that will discourage negative practice of transplant tourism. There is need for larger multicentre studies of organ tourism in Eastern Nigeria.
